# A Novel Computer-Aided Diagnostic System for Early Detection of Diabetic Retinopathy Using 3D-OCT Higher-Order Spatial Appearance Model

**DOI:** 10.3390/diagnostics12020461

**Published:** 2022-02-11

**Authors:** Mohamed Elsharkawy, Ahmed Sharafeldeen, Ahmed Soliman, Fahmi Khalifa, Mohammed Ghazal, Eman El-Daydamony, Ahmed Atwan, Harpal Singh Sandhu, Ayman El-Baz

**Affiliations:** 1 Bioengineering Department, University of Louisville, Louisville, KY 40292, USA; mohamed.elsharkawy@louisville.edu (M.E.); a.sharafeldeen@louisville.edu (A.S.); ahmed.soliman@louisville.edu (A.S.); fahmi.khalifa@louisville.edu (F.K.); harpal.sandhu@gmail.com (H.S.S.); 2 Electrical and Computer Engineering Department, College of Engineering, Abu Dhabi University, Abu Dhabi 59911, United Arab Emirates; mohammed.ghazal@adu.ac.ae; 3 Faculty of Computers and Information, Mansoura University, Mansoura 35516, Egypt; emane_daydamoni@mans.edu.eg (E.E.-D.); ahmed.atwan@nbu.edu.sa (A.A.)

**Keywords:** 3-D optical coherence tomography (3-D OCT), diabetic retinopathy (DR), neural network (NN), majority voting, computer-aided diagnosis (CAD), Markov–Gibbs random field model (MGRF)

## Abstract

Early diagnosis of diabetic retinopathy (DR) is of critical importance to suppress severe damage to the retina and/or vision loss. In this study, an optical coherence tomography (OCT)-based computer-aided diagnosis (CAD) method is proposed to detect DR early using structural 3D retinal scans. This system uses prior shape knowledge to automatically segment all retinal layers of the 3D-OCT scans using an adaptive, appearance-based method. After the segmentation step, novel texture features are extracted from the segmented layers of the OCT B-scans volume for DR diagnosis. For every layer, Markov–Gibbs random field (MGRF) model is used to extract the 2nd-order reflectivity. In order to represent the extracted image-derived features, we employ cumulative distribution function (CDF) descriptors. For layer-wise classification in 3D volume, using the extracted Gibbs energy feature, an artificial neural network (ANN) is fed the extracted feature for every layer. Finally, the classification outputs for all twelve layers are fused using a majority voting schema for global subject diagnosis. A cohort of 188 3D-OCT subjects are used for system evaluation using different *k*-fold validation techniques and different validation metrics. Accuracy of 90.56%, 93.11%, and 96.88% are achieved using 4-, 5-, and 10-fold cross-validation, respectively. Additional comparison with deep learning networks, which represent the state-of-the-art, documented the promise of our system’s ability to diagnose the DR early.

## 1. Introduction

Pathology of the eye is one of the most serious medical concerns as the eye is one of the most important sensory organs in the body. Blindness can occur from a variety of eye diseases, such as diabetic retinopathy (DR), age-related macular degeneration (AMD), and glaucoma. It is unfortunate that these conditions are silent in their early stages, and can only be diagnosed by regular eye exams. This manuscript presents a characterizing study of DR, a serious progressive vascular and neurodegenerative disease resulting in retinal cell damage without causing any visible visual impairment and can be difficult to detect at first. An uncontrolled blood sugar level can result in DR, which results in progressive damage to the retina and impaired vision. It is therefore necessary to detect DR early in order to prevent retinal damage that could lead to blindness in the future. According to Saeedi et al. [1], in 2019, approximately 463 million people globally have DR, 31 million of whom are in the United States. According to estimates, this number will grow to 578 million (34.4 million in U.S.) through 2030 and 700 million (367 million in U.S.) through 2045. Moreover, according to the Centers for Disease Control and Prevention (CDC) [2], the incidence of DR has been reported at 4.1 million in the U.S. In addition, approximately $500 million is spent by the U.S. each year due to diabetes-related blindness [2]. Approximately 899,000 Americans have visual impairments. Therefore, it is crucial to detect DR at an early stage. Once the patient appears to have symptoms, they often develop with a progressive disease that can lead to numerous and possibly blinding structural complications, like tractional retinal detachment, proliferative diabetic retinopathy (PDR), and neovascular glaucoma. Therefore, detecting DR early is, consequently, crucial in order to avoid permanent blindness.

Clinical management of DR first involves improved systemic control of blood pressure and blood glucose. Beyond systemic control, there are three treatment options for diabetic retinopathy that are routinely employed in the clinic: intravitreal anti-VEGF injections, laser photocoagulation, and vitreoretinal surgery. The first two are the mainstays of therapy, as they can be performed easily and routinely in the setting of an outpatient clinic. The third, vitreoretinal surgery (also called a vitrectomy), is reserved for the most advanced cases of proliferative vitreoretinopathy, which have been complicated by dense vitreous hemorrhages and/or tractional retinal detachment.

Different imaging modalities are used for clinical follow-up, including Fundus Photography (FP), Fluorescein Angiography (FA), Optical Coherence Tomography (OCT), and Optical Coherence Tomography Angiography (OCTA). Every modality has its own advantages when it comes to revealing the retina. In the last few years, there have been many researchers using retinal imaging techniques to detect diseases that may cause vision loss. Some of these works utilize FP and FA, which consume a considerable amount of time to discern morphological changes of the optic disk and exudates using manual screening.

OCT has become one of the most useful modalities for ophthalmologists to detect many diseases, such as DR. In this modality, cross-sectional images are produced without any invasive procedures and produced a volumetric view of the retina using interferometry to visualize microscopic changes such as macular edema. In the following section, an overview of the literature image-based CAD systems for ocular diseases diagnosis using OCT images is given.

### Related Work

There has been some CAD systems works on detecting DR using fundus photographs and Fluorescein Angiography. For example, Foeady et al. [3] examined the ability of a support vector machine (SVM) classifier to accurately grade DR in fundus photographs. A morphological operation and median filter were applied to improve the image in their system. Their next step was to construct a gray-level co-occurrence matrix to extract statistical features e.g., energy, correlation, homogeneity, and contrast. A SVM was then used to classify these features, and they reported that the system was accurate at 82.35%. Another system by the authors of [4] is used to diagnose the DR. Using fuzzy image preprocessing combined with Machine Learning (ML) algorithms, a detection system for DR is presented that uses color fundus images.

In addition, there has been considerable success in exploiting OCT for detecting retinal diseases like DR, AMD, and DME. For example, Sandhu et al. [5] implemented a CAD system utilizing the two modalities, e.g, OCT and OCTA, to detect and grade DR. They also added demographic data for DR patients and fused it with the features extracted from OCT and OCTA images. Bernardes et al. [6] developed a CAD system using the OCT images to make grading for DR. They used OCT histogram information in their system as a feature extraction from OCT images. Then, they fed these features to SVM to classify DR. An experimental validation based on leave-one-subject-out results showed that 66.7% of them were correctly classified. However, they were very inaccurate in their result when comparing with other experiments. A different system is described in [7], the authors of which developed a CAD system to diagnose glaucoma using the OCT images. They used convolutional neural network (CNN) to extract the features and used the softmax layer to classify the OCT images. Then, they evaluated their system using the area under the ROC Curve. The system had an AUC of 94%. There are other works that use OCT to diagnose DR in [8,9,10,11].

OCTA and OCT, which are noninvasive techniques, have been used in other investigations to identify retinal disorders because they provide cross-sectional and volumetric views of retinas and blood vessels, respectively. Alam et al. [12] developed an algorithm for detecting DR and grading DR using a classification system based on SVM in OCTA scans. OCTA scans were analyzed to determine six features, including blood vessel caliber, blood vessel curvature, foveal avascular zone size, vessel perimeter index, blood vessel density, and irregularity of the foveal avascular zone contour. A fusion of all features was presented as well as the results of every feature individually. Blood vessel density was the most accurate feature, but fusion of the features gave the best results. As reported, the fusion features were more diagnostic for normal compared with DR and normal compared with different grades of DR, with accuracy 94.41% and 92.96%, respectively. A CAD system was developed [13] using an extension of the CAD system from their prior study [14], the authors established a methodology for diagnosing DR using the 3D-OCT volume by the local binary pattern (LBP), histogram of oriented gradients (HOG). They used the principal component analysis (PCA) to reduce the dimensions of features. Each feature is separately fed into different classifiers. The best classifier used for the histogram of LBP using PCA is SVM with linear kernel which achieves sensitivity and specificity of 87.5% and 87.5%, respectively. Due to lack of layer segmentation and poor performance, this work has some shortcomings. Ibrahim and colleagues [15] have presented a CAD system which utilizes a pretrained deep learning method based on the VGG16 convolutional neural network to diagnose DM, CNV, and drusenoid disorders in a 3D-OCT volume by adding the features extracted from the deep neural network to hand-crafted features extracted from the ROI. Ghazal and colleagues [16] explained how to apply CNN CAD to analyze OCT B-scans to detect DR. First, an average B-scan is made up of five areas, including nasal, distal nasal, central, distal temporal, and temporal. Second, a total of seven distinct CNNs were trained, each based on a region plus two transfers based on only the nasal and temporal regions. Last, to obtain the overall diagnosis, the seven CNN results were fed, individually or together, with two regions of analysis (nasal and temporal) and transfer learning, the performance of the established system has been reported to be 94%. CAD systems integrating the two modalities have also been utilized to diagnose DR grades in another study [5]. Clinical and demographic data are combined with the findings from the two modalities and input into a classification system, namely random forest (RF) classifier.

There have also been other works that used OCT with different outcomes [6,7,8,9,10,17,18,19,20,21,22,23,24,25,26,27,28,29,30].

The proposed CAD system integrates a segmentation method to segments the twelve retinal layers for each B-scan in a 3-D OCT volume. For this, the segmentation utilizes an adaptive shape prior knowledge. This is followed by extracting a novel texture feature (2nd-order reflectivity) that is derived from a Markov–Gibbs random field (MGRF) model where the second-order structure of image gray levels by treating each B-scan in the OCT volume as an instance of one MGRF. Finally, we construct a cumulative distribution function (CDF) of Gibbs energy values throughout each layer and use its nine deciles to create a vector descriptor for each layer. Layer-wise deciles features are then concatenated and an artificial neural network (ANN) is fed with these features for testing and training. The proposed system is evaluated and conducted using different validation techniques using a majority voting schema.

This contribution of the work presented in this manuscript can be described as follows:

- Each layer of the OCT is analyzed separately and is classified for local and individualized analyses. In the following step, the decision of the individual layers is combined into a global diagnosis.
- Our system integrates incorporates a 3D-MGRF model, which is in place of using a 1st-order reflectivity.
- To improve the descriptive power of the extracted features, a statistical approach is used (i.e., CDF percentile).
- The CDF percentile values are fed into an ANN to get the final diagnosis of the 3D OCT volume.

## 2. Method

**Figure 1:**
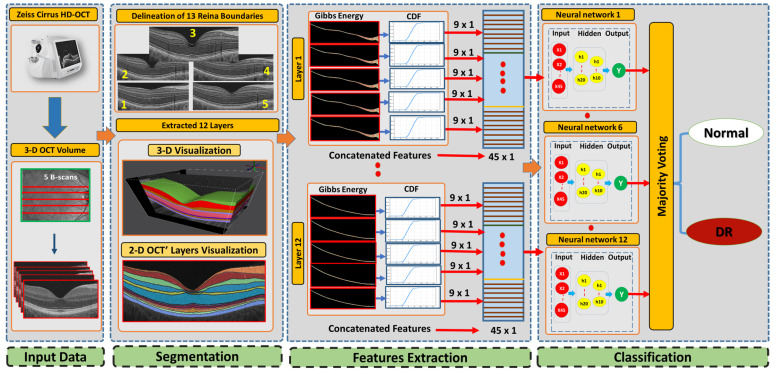
The framework of the proposed CAD system for DR diagnosis using 3-D OCT images.

This paper presents a new 3D-OCT CAD system based on a higher-order spatial appearance model and illustrates in Figure 1. The developed CAD system is divided into three phases. Using our developed segmentation method [31] that employs an appearance-based approach, we segment the 3D-OCT into 12 layers in the first phase. The second phase distinguishable higher order reflectivity feature are extracted. In the third stage, classification of individual layers based on the extracted feature are done as well as applying a majority voting for the classification layer’s outputs to get the global diagnosis. In the following subsections, we describe the 3D-OCT segmentation method and the novel feature extracted from segmented retinal layers.

### 2.1. 3D-OCT Volume Segmentation

In this approach, the first slice through the middle of the fovea is used to segment macular OCT. To do so, MGRF modeling was implemented to identify the 12 retinal layers surrounding the fovea. An atlas of the retina transmits the expected shape, relative contrast, and uniformity encoded in it. A 3D-OCT volume is totally segmented once the data from the central section are transmitted through the superior and inferior B-scans with respect to the middle slice. Using the segmentation method, the following steps are performed:

- Step 1: The B-scans are matched to a shape database built by the expert. The database contains manually segmented foveal B-scans representing normal and diseased retinas.
- Step 2: The second step is to divide the region between the vitreous and choroid into twelve distinct segments based on the interaction model for shape, intensity, and spatial interactions.
- Step 3: In the third step, the unprocessed B-scans are used as prior shape models in the process of segmenting each segmented B-scan.
- Step 4: The prior shape models are refined to obtain the final segmentation

**Figure 2:**
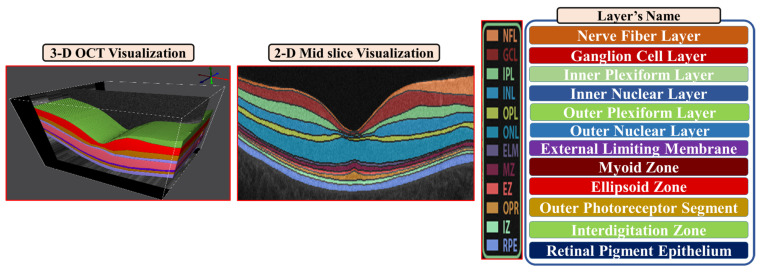
A 3D-OCT visualization of the layer segmentation (left). On the right, a description of twelve layers starting from layer one (the nerve fiber layer (NFL)) and ending with layer 12 (retinal pigment epithelium (RPE)).

In Figure 2, a 3D-OCT visualization for the layer segmentation is obtained as well as we describe the twelve layers starting from layer one (the nerve fiber layer (NFL)) and ending with layer 12 (retinal pigment epithelium (RPE)).

**Figure 3:**
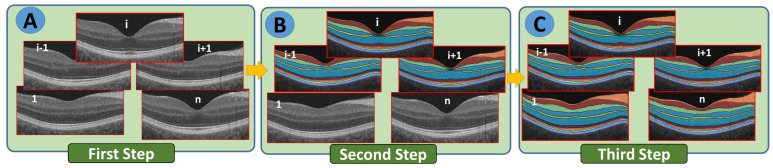
An explanation of the segmentation process shows how the B-scan 3D OCT images are segmented beginning at the macula mid slice (*i*) and moving in two directions (**A**–**C**).

An entirely new approach was developed to implement single-slice segmentation through the entire macular 3D-OCT volume. This approach uses an adaptive shape prior that considers reflectivity values in addition to mapped voxel locations. A shape prior is used that includes mapped voxel locations in addition to reflectivity values. Models that incorporate prior shape information do not take into account visual appearance when generating probabilistic maps. According to the first-order adaptive shape-intensity model, segment labels are propagated primarily onto regions of adjacent B-scans with similar appearance (OCT reflectivity) to the labeled region in the reference slice. In Algorithm 1, we describe the segmentation process propagation. In both directions, we describe in Figure 3 the process of segmenting the whole OCT B-scan volume starting with the mid slice. The macular OCT is centered on the fovea; thus, the middle slice is a cross-section of the fovea. Our segmentation approach is based on segmenting the fovea B-scan based on our previous 2D segmentation work and extending that to 3D segmentation (the remaining 4 slices), where the center B-scan is adapted as a patient-specific atlas in a segmentation propagation scheme. In the next steps, we illustrate the segmentation process with respect to a volume of 5 B-scans from 3-D OCT images layered in 12 layers: (i) by using a joint-MGRF algorithm, the 12 retinal layers in the 3rd OCT slice through the fovea are labeled; (ii) the 12-layer segmentation of slice 3 can be propagated to slices 2 and 4 as a result of the patient-specific atlas of slice 3; and (iii) the segmentation of slices 2 and 4 can then be propagated to slices 1 and 5 using the patient-specific atlas as a guide.
**Algorithm 1:** Prior shape propagation algorithm **Require:** Macular OCT comprising *K* B-scans **Require:** Segmentation of B-scan *n* labeling 12 retinal layers1:**for**i←n−1,…, 1**do**2: Warp B-scan *i* to align with B-scan i+1 using non-rigid registration3: **for all** pixel *v* in B-scan *i* **do**4:  Let $v^{\prime }$ be the corresponding pixel in B-scan i+1 according to the non-rigid mapping5:  Let *w* be a $K_{1i}\times K_{2i}$ window centered on $v^{\prime }$6:  Identify pixels in *w* with gray level intensity within a given threshold τ of that of *v*7:  If no pixels meet the threshold criterion in step 5, expand *w* and repeat until in-range gray levels are found or a pre-set maximal window size is exceeded8:  Calculate the posterior probability *v* belongs to a retinal layer according to the incidence of that layer’s label among pixels in *w* satisfying the threshold criterion9:  Assign *v* the segment label with the highest corresponding probability10: **end for**11:**end for**12:**for**i←n+1,…, K**do**13: Warp B-scan *i* to align with B-scan i−1 using non-rigid registration14: Repeat steps 4 through 915:**end for**

### 2.2. Feature Extraction

**Figure 4:**
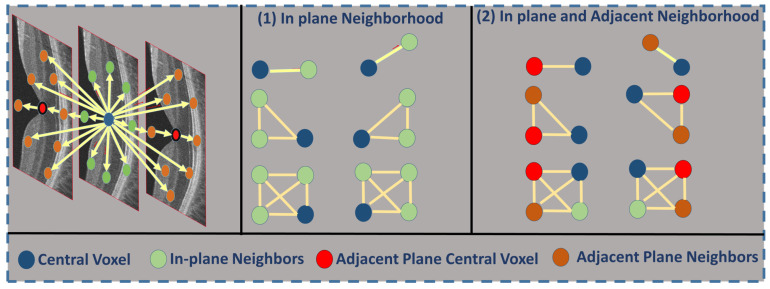
A graphic representation of 26 neighborhood voxels (left panel) for the higher-order 3D-MGRF model; and examples of different order cliques of center voxel (blue) and its neighbors in the same plane (middle panel) and adjacent planes (right panel).

The second stage in our developed method ascertains the higher-order reflectivity feature from the segmented retina layers. Each B-scan in the OCT volume is modeled as one instance of the MGRF in order to model the second-order structure of gray levels in images. An algorithm is used to generate random fields based on interactions among pixels within a fully connected neighborhood, or clique (see Figure 4). Our proposed method utilizes a eight-neighbors translation-invariant system for the 3D-MGRF, N={(0,1),(0,−1),(1,0),(−1,0),(1,1),(1,−1),(−1,−1),(−1,1)}.

Let us define the notation as follows:

- g:R→Q is a grayscale image on the discrete domain R⊂Z×Z with pixel values in Q={0,…,Q−1}.
- $N=\{\left(\xi_{i},\eta_{i}\right),i=1,\ldots ,n\}$ is a set of (x,y)-offsets specifying the pairwise neighborhood system.
- *C* is the graph of pixel interactions on *R*; the neighborhood system for pixel (x,y)∈R is the set of pairs C(x,y)={{(x,y),(x+ξ,y+η)},(ξ,η)∈N}.
- $V_{i}:Q\times Q\to R$ is the Gibbs potential associated with neighborhood $\left(\xi_{i},\eta_{i}\right)$.

With these preliminaries, the second-order MGRF on *R* is specified by its Gibbs probability distribution function:

(1) $$P\left(q\right)=\frac{1}{Z}\exp\left(\left|R\right|\sum_{i=1}^{n}\sum_{q^{\prime }=0}^{Q-1}V_{i}\left(q,q^{\prime }\right)F_{i}\left(q,q^{\prime }\right)\right)$$

where *Z* is the partition function, |R| is the cardinality of *R*, and $F_{i}$ is the bivariate empirical probability distribution, i.e., scaled gray level co-occurrence matrix, over neighborhood family *i*. Determination of the MGRF model reduces to estimation of the potentials $V_{i}$ in Equation (1). This is achieved by using the analytical maximum likelihood estimator for the 2nd-order Gibbs potentials introduced in [32],

(2) $$V_{i}\left(q,s\right)=\rho_{i}\left(F_{i}\left(q,s\right)-f_{i}\left(q\right)f_{i}\left(s\right)\right),$$

where $f_{i}\left(q\right)=\frac{1}{Q}\sum_{q^{\prime }=0}^{Q-1}F_{i}\left(q,q^{\prime }\right)$ is the gray-level marginal distribution. The Gibbs potentials are calculated on a per-layer basis. Empirical gray-level distributions are calculated within each B-scan, then averaged across B-scans to obtain the final $F_{i}$ and $f_{i}$ to calculate Gibbs potentials for that layer throughout the OCT volume. Then, for each retinal layer *l* in the OCT images there is a corresponding vector of Gibbs energies describing that layer’s texture,

(3) $$E_{l,i}=\sum_{q=0}^{Q-1}\sum_{q^{\prime }=0}^{Q-1}F_{l,i}\left(q,q^{\prime }\right)\left(F_{l,i}\left(q,q^{\prime }\right)-f_{l,i}\left(q\right)f_{l,i}\left(q^{\prime }\right)\right)$$

**Figure 5:**
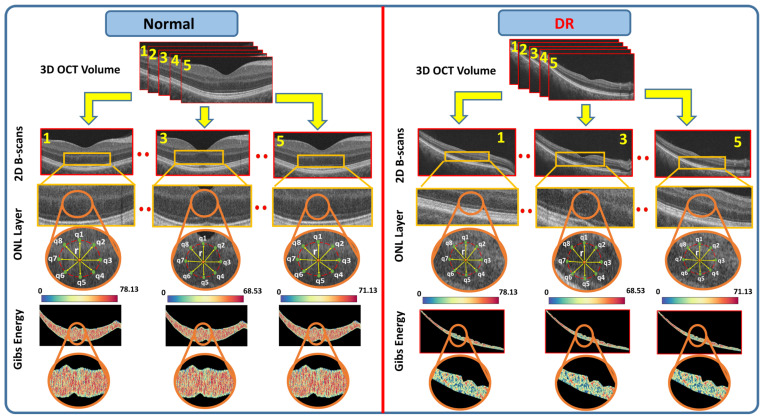
A color-coded illustration for the higher order reflectivity feature (Gibbs energy) extracted from segmented layer (ONL) for a healthy case (left panel) against a DR subject.

A comparison of the healthy case and DR case at the ONL layer is illustrated with the possible values of the estimated Gibbs energy in Figure 5. At the end, to comprehensively describe each layer’s Gibbs energy values, a CDF is constructed for the nine deciles and a vector descriptor is created (Algorithm 2).
**Algorithm 2:** Extracting the feature vector using 3D-MGRF **Require:** *g*, *N*, *C* as defined above **Require:** segmentation M:R→L1:Initialize empty feature vector *U*2:**for all**l∈L, *l* a retinal layer **do**3: Initialize $F_{l}=0$4: **for** B-scan k=1,…,n **do**5:  Let $S_{k,l}=\left\{\left(x,y\right)|M_{k}\left(x,y\right)=l\right\}$6:  Let $C_{k,l}$ be the restriction of $C_{k}$ to $S_{k,l}$7:  Let $F_{k,l}$ be the bivariate gray level distribution, as in Equation (1), of $g_{k}$8:  $F_{l}\leftarrow F_{l}+F_{k,l}$9: **end for**10: $F_{l}\leftarrow F_{l}/n$11: Calculate Gibbs energies of all pixels in layer *l* using Equation (3)12: Let $U_{l}=\left(E_{\left(10\right)},E_{\left(20\right)},\ldots ,\;E_{\left(90\right)}\right)$ where $E_{\left(p\right)}$ is the $p^{\mathrm{th}}$ percentile of Gibbs energies13: $U\leftarrow (U,U_{l})$14:**end for**

### 2.3. Classification System

In the third stage of our developed approach, we build a classification system based on an ANN to diagnose DR. The developed classifier involves two stages to obtain the final diagnosis for DR. First, we trained an ANN for layer-wise classification for the 3D-OCT volume. Twelve ANNs were fed with the concatenated features for each layer in all five scans of 3D-OCT. Second, a majority voting (MV) scheme was applied to the output of the ANNs to produce the global subject diagnosis. To make these features more meaningful to represent the layer, a CDF of feature values was constructed. Algorithm 3 outlines the essential process for training the ANN.
**Algorithm 3:** ANN trainingInitialization: Random weights with randomly selected numbers from normal distribution are assigned to the designed ANN network.Feed-Forward: All the hidden layers’ neurons and output layers’ neurons are calculated and moved forward in the network.Backpropagation: using the stochastic gradient decent optimization method, utilize backpropagation to calculate weights for the proposed ANN network.Replicate procedures 2 and 3 until the ANN weights do not change substantially.

To optimize network architecture, hyperparameter tuning is performed including varying the hidden layer number (1 or 2) and the size of the hidden layers (5–20 neurons, inclusive). The best configuration for the ANN following tuning were two hidden layers with 20 and 10 neurons, respectively. As for the activation function for the hidden layer and the output layer, it is a hyperbolic tangent for the hidden layer and softmax for the output layer. We select the best one from sigmoid, tangent, softmax, and rectified linear activation function.

## 3. Experimental Results

In this study, we tested our developed CAD system using 188 3D-OCT volume. The dataset consists of 100 healthy OCT scans and 88 DR Scans. Zeiss Cirrus HD-OCT 5000 [33] was used to collect the OCT data to do different experiments, which were acquired at the University of Louisville hospital. All patients were informed of the study’s purpose and provided their consent after approval of the research protocol by the University Institutional Review Board (IRB). For each OCT volume, we acquired for each eye (right or left) 5 OCT image scans. The dimensions for each collected volume that were utilized in this paper is 1024 × 1024 × 5 voxels, 8.80 × 1.96 × 500.00 mm${}^{3}$, and 9.01 × 2.00 × 2.50 mm${}^{3}$. The range of the ages were from 21 to 84 years. The medical retina specialists performed manual segmentation. Two specialists reviewed every image and came to an agreement on segmentation for each image, thus minimizing the risk of human error. There were twelve distinct layers in three-dimensional surface volumes separated by 13 boundary lines. An OCT scan of the macula was conducted in every eye on all diabetic subjects regardless of ocular history in a clinical setting. The scan had a minimum signal strength of 7/10. Physicians with sub-specialization in retinal disease determined the degree of disease and the clinical grade of the patient. Each eye was examined with dilated fundus examinations to determine whether there was significant retinopathy.

Since we have two classes in our dataset, e.g., healthy and DR, we evaluated our system based on each class outcomes. Thus, we used three evaluation metrics to determine quantitative efficiency, i.e., accuracy, sensitivity, and specificity. For each class, we calculate true positive (*TP*), false positive (*FN*), true negative (*TN*), and false negative (*FN*). Then, we calculated the three evaluation matrices as follows:

(4) $$Specificity=\frac{TP}{TP+FP}$$

(5) $$Sensitivity=\frac{TP}{TP+FN}$$

(6) $$Accuracy=\frac{TP+TN}{TP+FP+TN+FN}$$

The performance of the developed system was evaluated using cross-validation of K-folds. There were various scenarios used to validate the system: 4-folds, 5-folds, and 10-folds. The different matrices mentioned above accuracy, sensitivity, and specificity have been used to measure these quantitative performances for the proposed system. In addition, we divided the dataset into train set and test set. We used the train set to train and tune the model parameters to get the best hyperparameters for ANN. Furthermore, We split the dataset into 70/30 percent (train/test). The results are summarized in Table 1. According to this table, for the layer classification using *10-fold* cross-validation, the developed system accuracies for NFL, GCL, IPL, INL, OPL, ONL, ELM, MZ, EZ, OPR, IZ, and RPE were 95.69%, 89.69%, 88.78%, 89.60%, 87.87%, 91.93%, 80.75%, 76.47%, 77.11%, 76.80%, 86.24%, and 90.21%, respectively. In addition, the accuracy of our developed system using the majority voting is 96.88%, which gave the best value compared to *4-fold* and *5-fold* cross-validation and the testing dataset. While the accuracies of the majority voting for the *4-fold*, *5-fold*, and test set were 90.56%, 93.11%, and 87.50%, respectively.

It would be unfair to compare the proposed system with those systems in the literature that use CNN and/or FP to classify DR because these environments are too different from the one proposed. To that end, we evaluated our proposed system against various ML methods (i.e., SVM with linear and radial basis function (rbf) kernel, logistic regression (LR), Naive Bayes (NB), KNN, random forest (RF), and decision tree (DT)). The results of the comparative experiments are presented in Table 2. As demonstrated in the table, our method has the highest accuracy of among the compared methods. For example, the accuracies using *10-folds* cross validation for SVM with linear, SVM with rbf, LR, NB, KNN, RF, and DT were 77.45%, 76.06%, 81.91%, 72.96%, 78.16%, 79.54%, and 81.91%, respectively. While comparing all of these ML algorithms with our developed system, the system gave the highest accuracy with 96.88% using 10-fold cross validation.

Moreover, we compared the performance of our proposed method with well-known deep learning techniques (AlexNet [34], ResNet50 [35], and Inceptionv3 [36]). Therefore, we used and tuned the hyperparameters for these pretrained CNNs to get comparative results with the proposed system. Using the grid search for the optimizer (Adam and stochastic gradient descent with momentum (SGDM)) as search spaces, we tuned and selected the best hyperparameters for all pretrained CNNs. Moreover, for every 5 iteration, the learning rate decreased by a factor of 10 from 0.001. To find the best-fit training model, we imposed a maximum number of epochs of 500 and monitored the validation loss. In Table 3, the accuracy of our developed method gave the best accuracy compared with the well known pretrained CNN ResNet50, AlexNet, Inceptionv3 which resulted accuracies of 94.90%, 92.33%, and 95.60%, respectively, using *10-folds* cross validation.

**Figure 6:**
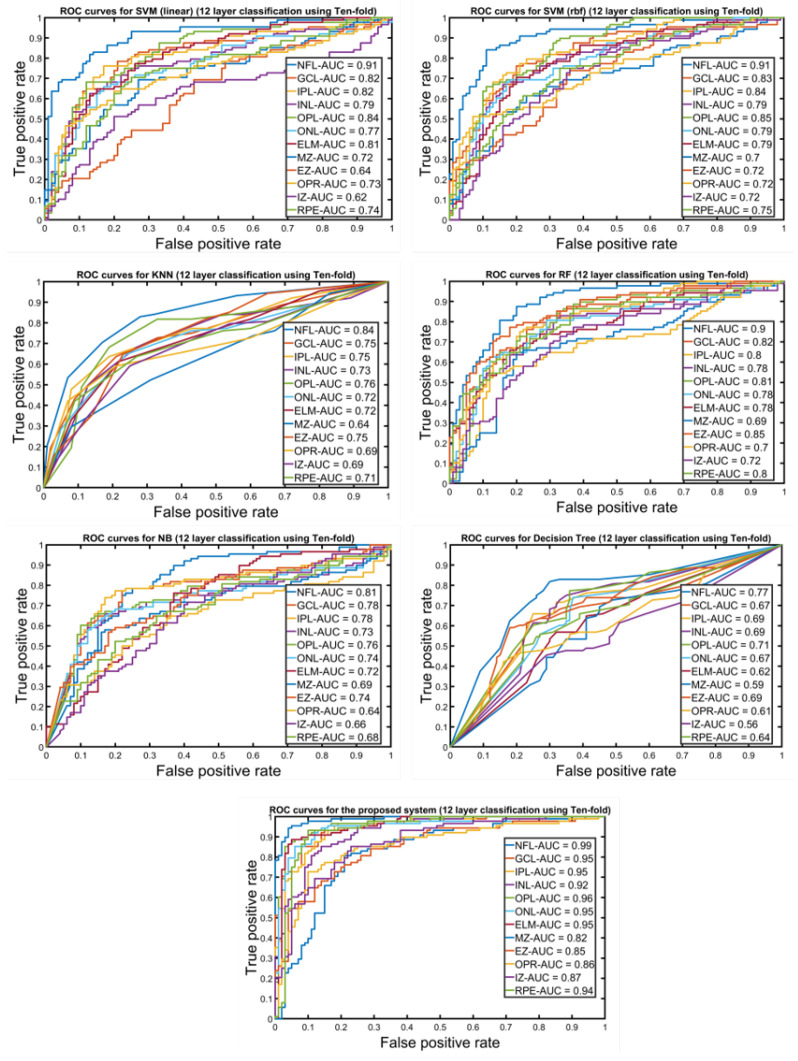
An illustrative ROC curves for the classification of 12 layers for the proposed system in comparison with other machine learning approaches using ten-fold cross-validation.

Furthermore, to demonstrate the reliability of the developed system, the receiver operating characteristics (ROC) for each layer is constructed. Figure 6 shows the ROC curves for all 12 layers’ classifications using the *10-fold* cross-validation for the proposed system against the other machine leaning techniques. According to the calculated AUC for all retina layer using *10-fold* cross-validation, our proposed system shows the highest values for all layers when we compared with the other ML techniques (i.e., SVM with linear and radial basis function (rbf) kernel, logistic regression (LR), Naive Bayes (NB), KNN, random forest (RF), and decision tree (DT)). Moreover, the AUC values of the proposed system for all retina layers gave 0.99, 0.95, 0.95, 0.92, 0.96, 0.95, 0.95, 0.82, 0.85, 0.86, 0.87, and 0.94 for NFL, GCL, IPL, INL, OPL, ONL, ELM, MZ, EZ, OPR, IZ, and RPE, respectively.

## 4. Conclusions and Future Work

As a result of this work, a new diagnostic OCT CAD system detecting DR early has been developed, allowing early intervention and the prevention of vision loss with the use of a more detailed 3D-OCT. The study included two grades of non-proliferative diabetic retinopathy (NPDR): mild and moderate. This study did not include eyes with macula edema. A low number of cases of severe NPDR and proliferative diabetic retinopathy also prevented the inclusion of those grades. The proposed system estimates a higher order spatial appearance model based on 3D-MGRF from automatically segmented 3D-OCT retina layers. The proposed system achieves a promising result when compared with state-of-the-art deep learning networks in addition to ML algorithms. The best accuracy was 95.70% when we used 10-fold cross-validation applied on the dataset. In order to improve this value of accuracy, we plan to add more features and analyze a larger number of images. In addition, we intend to study and investigate the potential enhancement of the proposed system combined with other scan modalities and clinical biomarkers in the near future. It is also possible to generalize this system to diagnose other eye conditions that damage the retinal layers and result in vision impairment.

## Figures and Tables

**Table 1:** The evaluation of the proposed CAD system for DR diagnosis. Note that Acc.: Accuracy, Sens.: Sensitivity, Spec.: Specificity.

Layer	Four Fold			Five Fold			Ten Fold			Test Set		
	Acc.	Sens.	Spec.	Acc.	Sens.	Spec.	Acc.	Sens.	Spec.	Acc.	Sens.	Spec.
**NFL**	91.25%	90.82%	92.95%	93.89%	94.24%	93.94%	95.69%	94.55%	96.69%	91%	89.45%	91.30%
**GCL**	79.88%	75.93%	88.47%	86.45%	86.23%	85.37%	89.69%	97.48%	81.93%	80.35%	88.23%	68.18%
**IPL**	80.81%	74.96%	87.98%	82.90%	81.51%	82.15%	88.78%	90.42%	87.70%	69.64%	70.58%	68.18%
**INL**	87.96%	85.70%	90.19%	90.75%	89.73%	90.98%	89.60%	87.15%	87.98%	85.71%	85.29%	86.36%
**OPL**	84.60%	84.19%	83.69%	92.36%	94.89%	87.96%	87.87%	91.83%	87.94%	80.35%	76.47%	86.36%
**ONL**	84%	82.99%	82.64%	74.80%	71.97%	80.19%	91.93%	90.84%	90.80%	82.14%	91.17%	68.18%
**ELM**	80.70%	78.36%	80.63%	84.87%	80.96%	87.60%	80.75%	81.69%	81.96%	76.78%	73.52%	81.81%
**MZ**	73.21%	74.97%	69.87%	74.60%	76.40%	71.60%	76.47%	78.41%	70.94%	66.07%	70.58%	59.09%
**EZ**	77.59%	77.32%	78.56%	77.60%	78.90%	76.85%	77.11%	76.56%	78.13%	71.42%	76.47%	63.63%
**OPR**	75.12%	81.32%	69.74%	77.98%	78.87%	73.61%	76.80%	73.72%	84.68%	58.92%	94.11%	63.60%
**IZ**	75.56%	73.89%	73.77	72.87%	80.41%	67.35%	86.24%	82.95%	90.55%	67.85%	76.47%	54.54%
**RPE**	83.98%	80.69%	88.63%	87.12%	87.54%	86.90%	90.21%	86.65%	92.99%	75%	85.29%	59.09%
**Majority Voting**	**90.56%**	**98.63%**	**86.98%**	**93.11%**	**96.63%**	**86.98%**	**96.88%**	**97.89%**	**95.27%**	**87.50%**	**96.50%**	**82.39%**

**Table 2:** Comparison between the proposed system and other ML-based classification. Note that Acc.: Accuracy, Sens.: Sensitivity, Spec.: Specificity.

Classifiers	Four Fold			Five Fold			Ten Fold		
	Acc.	Sens.	Spec.	Acc.	Sens.	Spec.	Acc.	Sens.	Spec.
**SVM (Linear)**	77.66%	91%	62.50%	76.59%	92%	59.09%	77.45%	91.25%	64.12%
**SVM (rbf)**	75.53%	88%	61.40%	76.06%	89%	61.30%	76.06%	88%	62.50%
**Logistic Regression**	72.87%	86%	57.95%	78.19%	88%	67.05%	81.91%	91%	71.59%
**Naive Bayes**	78.72%	74.59%	86.36%	71.27%	92%	47.72%	72.96%	92.68%	52.48%
**KNN**	70.74%	92%	46.60%	77.12%	90%	62.50%	78.16%	91.97%	62.10%
**Random Forest**	78.19%	88%	67%	77.65%	89%	64.77%	79.54%	88.36%	66.17%
**Decision Tree**	72.87%	86%	57.95%	78.19%	88%	67.05%	81.91%	91%	71.59%
**Proposed System**	**90.56%**	**98.63%**	**86.98%**	**93.11%**	**96.63%**	**86.98%**	**96.88%**	**97.89%**	**95.27%**

**Table 3:** Comparison between the proposed system and other state-of-the-art deep learning approaches. Note that Acc.: Accuracy, Sens.: Sensitivity, Spec.: Specificity.

Classifiers	Four Fold			Five Fold			Ten Fold		
	Acc.	Sens.	Spec.	Acc.	Sens.	Spec.	Acc.	Sens.	Spec.
**ResNet50 [35]**	89.12%	97.75%	85.10%	90.91%	95.63%	85.14%	94.90%	92.8%	90.21%
**AlexNet [34]**	88.87%	95.74%	88.37%	89.65%	93%	85.90%	92.33%	93.54%	91.90%
**InceptionV3 [36]**	89%	96.34%	89.30%	90.53%	94.67%	84.30%	95.60%	96.33%	94.99%
**Proposed System**	**90.56%**	**98.63%**	**86.98%**	**93.11%**	**96.63%**	**86.98%**	**96.88%**	**97.89%**	**95.27%**

## Data Availability

Data are made available through the corresponding author upon a reasonable request.
